# Bridging the vitamin A and deworming coverage gap among underserved populations in India through government and civil society organization partnerships

**DOI:** 10.1186/s13690-024-01302-8

**Published:** 2024-05-20

**Authors:** Shilpa Bhatte, Jamie Frederick, Samantha Serrano, Clayton Ajello, Zaynah Chowdhury, Temjentsungla Jamir, Longri Kichu, Temsu Longchar, Ruchika Chugh Sachdeva, Neha Sareen, Amy Steets

**Affiliations:** 1 Vitamin Angel Alliance, Delhi, India; 2 Vitamin Angel Alliance, 6500 Hollister Ave Suite 130, Goleta, CA 93117 USA; 3 Department of Health and Family Welfare, Government of Nagaland, Kohima, Nagaland, India; 4 Bill and Melinda Gates Foundation, New Delhi, India

**Keywords:** Vitamin A, Deworming, Children under 5, Underserved populations, Equity tool, Lives saved tool

## Abstract

**Background:**

Vitamin A deficiency (VAD) is a major public health problem in India, where approximately 62% of children under five have low retinol levels (< 70 µmol/L). This study aims to (1) evaluate vitamin A supplementation (VAS) and deworming (VAS + D) coverage in Nagaland state through government and civil society organization (CSO) partnerships, (2) examine socio-demographic barriers and facilitators to VAS + D coverage, (3) examine associations between socio-demographic characteristics and source of VAS coverage (i.e., government vs. CSOs), and (4) estimate the impact of VAS on health outcomes due to increased coverage through government and CSO partnerships.

**Methods:**

A cross-sectional statewide coverage survey was conducted in Nagaland, India with 1,272 caregivers of children 6–59 months. Household socio-demographic data and VAS + D exposure variables were collected via quantitative survey. Univariate analyses were used to assess the associations between the independent and outcome variables; odds ratios were computed to measure the strength of the association at a significance level of < 0.05. The Lives Saved Tool (LiST) was used to estimate the impact of increased VAS coverage on child undernutrition, morbidity and mortality.

**Results:**

Most children (77.2%) received VAS in the past six months, with 28.1% receiving VAS in capsule form (provided primarily by CSOs) and 70.2% received VAS in syrup form (provided primarily by government). Total deworming coverage was 74.2%, with 43.5% receiving both VAS and deworming. Lower pre-school enrollment was a barrier to receiving VAS (47.4% not enrolled vs. 80.9% enrolled, *p* < 0.001). A barrier to receiving VAS + D was lack of knowledge of benefits (*p* < 0.001). Based on LiST modeling, increasing VAS coverage by 22% through CSOs resulted in an estimated 114 stunting cases averted, 25,017 diarrhea cases averted, and 9 lives saved in 2019 in Nagaland State.

**Conclusions:**

Government and CSO partnerships can reduce disparities in VAS coverage and decrease under-five child morbidity and mortality.

**Supplementary Information:**

The online version contains supplementary material available at 10.1186/s13690-024-01302-8.

**Table:** 

**Text box 1. Contributions to the literature**
• While scientific evidence and its corresponding literature demonstrates that vitamin A supplementation can increase child survival and prevent child morbidity and disability, gaps in universal vitamin A supplementation remain in India.
• This study contributes to the literature by showing that long-standing inequities in vitamin A supplementation and hence child health and survival can be bridged through innovative government and civil society collaboration and partnerships that aim to ensure that all children under 5 years of age in India are reached with essential health and nutrition services such as vitamin A supplementation and deworming.

## Background

Vitamin A deficiency (VAD) is a major public health problem in many low-and-middle-income countries (LMICs), including India, which is home to the largest number of vitamin A-deficient children in the world [1]. Around 62% of children under five in India have low retinol levels (< 70 µmol/L), which contributes to approximately 330,000 deaths annually [2]. Amidst the COVID-19 crisis, the number of children experiencing micronutrient deficiencies increased, due to disruption in health services and decreased access to food [3], making it increasingly important to ensure high coverage of vitamin A supplementation (VAS).

The World Health Organization (WHO) recommends that in the settings where VAD is a public health problem, high dose VAS (100,000 IU for children aged 6–11 months and 200,000 IU for children aged 12–59 months) should be universally provided [4]. Evidence demonstrates that VAS increases child survival, boosts the immune system, promotes physical growth, prevents blindness, mitigates the incidence of measles, and reduces under-five child mortality by 24% [5, 6]. Universal coverage of VAS is a cost-effective method of preventing child deaths [7, 8] and is considered one of 11 core maternal and child nutrition interventions for the prevention of child undernutrition and mortality [9].

In addition to a high prevalence of VAD, India has the world’s most acute parasitic worm problem, affecting 250 million children. India is endemic for soil-transmitted helminths [10], with a prevalence of 68% among children 1–19 years of age [11]. To reduce parasitic worm infections among preschool-aged children and thereby facilitate improved nutritional status, it is recommended by the WHO that deworming be paired with VAS [12]. Administering safe, effective deworming medication to children through platforms such as schools and *anganwadi* centers (i.e., government-funded child care centers) is a highly cost-effective intervention, due to its positive cost-effective impact on educational and economic outcomes [13].

Most countries provide VAS to children in the capsule form, except for India which provides VAS in syrup form. The national VAS protocol recommends VAS to children under five years for the prevention of blindness due to VAD and is implemented through biannual VAS campaigns in conjunction with routine immunization, or during village health sanitation and nutrition days (VHSND). To combat parasitic worm infections among preschool (*anganwadi*) and school-age children, the Government of India has adopted a single-fixed-day strategy called National Deworming Day (NDD), during which deworming (albendazole) tablets are administered to all children from 1 to 19 years of age through the platform of schools and *anganwadi* centers. In Nagaland State, these government programs improved coverage of VAS from 6.7 to 45.6% between 2005 and 2020 [14, 15], but deworming coverage decreased from 23 to 17% between 2005 and 2016 in children aged 6–59 months [14, 16]. However, there has been significant inter-state and inter-district variations in terms of coverage due to poor access to health services in especially underserved and hard-to-reach areas. When coordinated with national or local government, there is growing recognition that civil society organizations (CSOs) can play critical roles in improving quality and coverage of nutrition-specific interventions, such as VAS paired with deworming, through advocacy, promoting accountability, generating context-specific knowledge, and expanding service delivery to reach scale and increase equity among underserved populations [17].

The study’s objectives were to (1) evaluate the VAS and deworming (VAS + D) coverage in Nagaland state through government and CSO partnerships, (2) examine the socio-demographic barriers and facilitators to VAS + D coverage, (3) examine associations between socio-demographic characteristics and source of VAS coverage (i.e., government vs. CSOs), and (4) estimate the impact of VAS on health outcomes (i.e., child lives saved, undernutrition cases averted, and disease cases averted) due to increased coverage through government and CSO partnerships.

## Methods

### Study site

Located in a mountainous region bordering Myanmar, Nagaland State is geographically hard-to-reach and historically marginalized, receiving less support from the national government for health services compared with other states [18]. Vitamin Angels is a registered nonprofit focused on helping underserved populations in need -- specifically pregnant women, new mothers, and children under five -- gain access to lifesaving and life-changing nutrition solutions.

In 2005–2006, VAS coverage among children aged 9–59 months in Nagaland was low at 6.7% [14]. In 2011, Vitamin Angels partnered with a locally based CSO to deliver VAS (in capsule form) and deworming tablets to increase coverage. This resulted in an increase in vitamin A coverage from 6.7% [14] to 27% by 2015–2016 [16]. In 2012, Vitamin Angels expanded its partnership to provide VAS and deworming tablets to another CSO, and in 2016 provided product in coordination with the Nagaland State Government health services (who delivers VAS in syrup form), with the goal of further improving coverage.

### Study design and participants

Following the August 2018 child health week in Nagaland State, in which VAS and deworming distributions were held, a cross-sectional statewide survey was conducted to determine coverage of VAS among children aged 6–59 months of age and deworming among children 12–59 months of age. In each selected household, the primary caretaker was eligible for participation, and was interviewed after oral informed consent was taken for each eligible child. Data were collected on all children in the household 6–59 months of age.

### Sample size and sampling

The required sample size of respondents was calculated using the Neglected Tropical Disease Support Center’s Coverage Evaluation Survey Sample Builder (SSB) [19]. A total of 30 villages/wards were selected using probability proportional to size (PPS) sampling from 4 districts in Nagaland State. From these selected villages, a list of segments (a group of approximately 86 households) were prepared. In doing so, small villages with less than 86 households were merged with adjoining villages to form combined areas of 86 or more households to make segmentation more manageable in the field. The households of the combined areas were then equally divided into segments. The list of such segments served as a sampling frame, from which one segment was randomly selected. Finally, from the selected segments, households were approached for the interview as per criteria defined in WHO guidelines [20]. The required sample size of respondents was calculated using a sample size calculation considering the design effect, level of significance, desired precision, expected coverage and non-response rate.

A total sample of approximately 1,281 individuals were targeted to get statewide coverage estimates. To cover a sample of 1,281 respondents, a minimum of 2,562 households were estimated to be visited in sampled locations, as on average 0.5 members of each household have children less than 5 years of age, as estimated from Census 2011 data of Nagaland [21]. There were 2,984 households visited who had children in the eligible age group (6–59 months). Ultimately, 1,272 children were enrolled in the survey.

With 12 districts in the State of Nagaland, four strata were assigned based on each district’s geographic classification. One district from each of the strata was randomly chosen except for one stratum, where the aspirational district was purposively selected. Aspirational districts are defined by the Government of India via a ranking system based on indicators measuring health and nutrition, education, agriculture and water resources, financial inclusion and skill development, and basic infrastructure [22]. Aspirational districts rank lower on these social indicators than non-aspirational districts and are targeted by the government to improve their performance.

### Data Collection and Quality Assurance

Data collection was conducted by the Pramanit Karya India Private Limited (a local entity of Evidence Action in India) using the SurveyCTO software [23]. Study oversight was provided by Directorate of Health and Family Welfare, Government of Nagaland. To ensure the quality of data collected and minimize errors via use of automatic skip patterns and entry requirements, data collectors used computer-assisted personal interviewing (CAPI) devices. During fieldwork, supervisors revisited 10% of households to conduct a back check of the data. Timestamp and global positioning system (GPS) tracking were used at feasible locations to monitor the data collection of staff. Electronic data were reviewed for completeness daily.

### Measures

A survey instrument was developed by Pramanit Karya India Private Limited in conjunction with Vitamin Angels. The survey instrument included household-level socio-demographic questions, as well as questions measuring VAS and deworming coverage including source and location of interventions received, knowledge of the interventions and reported reasons for not receiving the interventions.

#### Receipt of VAS and/or deworming

Caregivers reported if their child(ren) 6–59 months received VAS and/or deworming in the last 6 months (yes/no). To determine the source of VAS, caregivers were asked if their child received VAS in syrup (government) or capsule form (CSO).

#### Socio-geographic characteristics

Child age, sex, school enrollment and maternal age, education and occupation were measured. Child age was categorized as 6–11 months and 12–59 months. Child school enrollment status was dichotomized as no enrollment or enrolled in school (including *anganwadi* centres or other). Mother’s age was categorized as 22 years old or lower, 23–29, and 30 or higher. Mother’s education was dichotomized as less than high school or high school or above, and mother’s occupation was dichotomized as not working or working. The purposefully selected aspirational district was classified as such, and the remaining three districts were classified as ‘non-aspirational.’. In addition, a wealth index called the EquityTool, developed by Metrics for Management, was applied to measure the relative wealth of households interviewed. The EquityTool is a short, country-specific questionnaire assessing types of consumer goods owned and housing characteristics such as source of drinking water and toilet facility type. When implemented, the tool provides a percentage of respondents in each national wealth quintile (1 = lowest wealth quintile, and 5 = highest wealth quintile). The scores are derived using principal component analysis [24]. Of the 12 questions in the Equity Tool, 11 were asked in the coverage survey. For the question about roof material that was not asked, all study participants were defaulted to the higher response option, ‘RCC/RBC/Cement/Concrete,’ per guidance from Metrics for Management.

### Statistical analyses

Descriptive statistics were used to explore the distribution, frequencies, means and standard deviations (SD) for study variables. Univariate logistic regressions were conducted to examine VAS and deworming coverage, as well as to compare VAS coverage by syrup versus capsule form. Caregiver knowledge of VAS and deworming was examined with descriptive analyses and univariate logistic regressions to examine the relation between knowledge of benefits and the receipt of VAS and deworming. We adjusted for the survey design by adjusting standard errors using the linearized Taylor series method using the SVY suite on Stata 17.0 software. All statistical analyses were conducted using Stata 17.0 software [25].

Finally, the Lives Saved Tool (LiST) was used to estimate changes in cause-specific mortality and morbidity based on annual increased coverage in VAS [26]. Using the subnational wizard in the LiST, two projections were developed in the LiST to model the scale-up of coverage of vitamin A from 2018 to 2019: a baseline projection using the preprogrammed vitamin A coverage value (25.6%) and the coverage of Nagaland government without CSO intervention (54.9%), and a coverage survey projection using the preprogrammed vitamin A coverage value (25.6%) and the coverage of Nagaland government with CSO intervention included (76.9%). Given these assumptions, we estimated additional child lives saved, stunting cases averted, and diarrhea cases averted by vitamin A intervention in Nagaland state in 2019.

## Results

### Characteristics of study participants

Child, maternal and household characteristics are described in Table 1. Of the 1272 children included in this sample, about half were female, with most falling within 12–59 months of age (87%) and most enrolled in school or an *anganwadi* center (89%). About half the mothers were between 23 and 29 years of age, with 40% older than 29 and 9% younger than 23. About half the mothers had a high school education or above (51%), and more than half were working (60%). The majority of households fell within the third wealth quintile (46%), with about a third in the lower two quintiles (33%) and 21% in the higher two quintiles. Finally, 84% of the study population resided in non-aspirational districts while 16% resided in the aspirational district of Nagaland. A comparison of the children retained in the analysis with those excluded can be found in Supplementary Table 1.

### VAS and deworming coverage

**Table 1:** Baseline child, maternal and household characteristics of enrolled children (*n* = 1272)

Child characteristics	*n*	%
**Child sex**		
Female	659	51.8
Male	613	48.2
**Age**		
6–11 months	171	13.4
12–59 months	1101	86.6
**School enrollment status**		
No enrollment	141	11.1
Enrolled in school (i.e. *anganwadi* or other)	1131	88.9
***Maternal characteristics***		
**Mother’s age (years)**		
22 or lower	294	9.0
23–29	1650	50.7
30 or higher	1311	40.3
**Mother’s education**		
Less than high school	1614	49.5
High school or above	1644	50.5
**Mother’s occupation**		
Not Working	1311	40.2
Working	1947	59.8
***Household characteristics***		
**Wealth Index Quintile**		
Quintile 1 (Lowest)	87	2.7
Quintile 2	975	29.9
Quintile 3	1497	45.9
Quintile 4	606	18.6
Quintile 5 (Highest)	93	2.9
**District**		
Aspirational district^21^ (*n* = 1)	525	16.1
Non-aspirational districts (*n* = 3)	2733	83.9

Of the 1,272 children included in this sample, those who responded ‘don’t know/don’t remember’ to receiving vitamin A in the last 6 months were dropped from the analysis, leaving 1,198 participants. Most children received vitamin A in the past six months (77%), of which 70% received VAS in syrup form provided by government, 28% in capsule form provided by a CSO, 1% received both forms and 0.7% did not recall what form of VAS was received. The total coverage of deworming among eligible children (i.e., children *≥* 12 months of age, *n* = 1,177) was 74%, with 44% receiving both VAS and deworming (Table 2).

**Table 2:** Coverage of vitamin A (*n* = 1198) and deworming (*n* = 1177) among children ages 6–59 months in Nagaland^*^

Coverage	*n*	%
Received vitamin A	925	77.2
Received deworming	873	74.2
Received vitamin A and deworming	233	43.5
***Among those who received vitamin A*** ^***†***^	925	
By syrup (government)	649	70.2
By capsule (CSO)	260	28.1
By capsule and syrup (government and CSO)	10	1.1
Don’t know	6	0.7

^*^Those who responded “don’t know” to receiving vitamin A or deworming were excluded from n

^†^The syrup form of VAS is provided primarily by the government, and the capsule form of VAS is provided primarily by the CSOs

### Sociodemographic barriers and facilitators to coverage

Of the 925 children who were reported to have received VAS, most were enrolled in school (81%, *p* < 0.001) and were between 12 and 59 months of age (81%, *p* < 0.001). On average, around 80% of children of each quintile received VAS, and around 77% of children from both aspirational and non-aspirational districts received VAS. Of those whose children received VAS or deworming, most caregivers reported knowing the benefits of VAS (93%) and deworming (88%), respectively (*p* < 0.001). Children of caregivers who knew at least one benefit of VAS were more likely to receive VAS as compared to those who knew no benefits of VAS (*p* < 0.001). Deworming followed the same trend; children of caregivers who knew at least one benefit of deworming were more likely to receive deworming as compared to those who knew no benefits of deworming (*p* < 0.001) (Table 3).

**Table 3:** Univariate associations^*^ between demographic variables and vitamin A supplementation (VAS) among children aged 6–59 months in Nagaland (*n* = 1198)

	Did not receive VAS (*n* = 273)		Received VAS (*n* = 925)		
Child characteristics	*n*	%	*n*	%	*p*-value
**Child sex**					
Female	138	22.4	479	77.6	0.713
Male	135	23.2	446	76.8	
**Age**					
6–11 months	79	50.0	79	50.0	< 0.001
12–59 months	194	18.6	846	81.4	
**School enrollment status**					
No enrollment	70	52.6	63	47.4	< 0.001
Enrolled in school (i.e. *anganwadi* or other)	203	19.1	862	80.9	
***Maternal characteristics***					
**Mother’s age (years)**					
22 or lower	40	37.7	66	62.3	0.444
23–29	119	19.5	490	80.5	
30 or higher	114	23.7	368	76.4	
**Mother’s education**					
Less than high school	164	27.5	433	72.5	0.086
High school or above	109	18.1	492	81.9	
**Mother’s occupation**					
Not Working	114	24.1	358	75.9	0.550
Working	159	21.9	567	78.1	
***Household characteristics***					
**Wealth Index Quintile**					
Quintile 1 (Lowest)	4	16.0	21	84.0	0.771
Quintile 2	78	21.8	279	78.2	
Quintile 3	127	23.1	423	76.9	
Quintile 4	59	25.9	169	74.1	
Quintile 5 (Highest)	5	13.2	33	86.8	
**District**					
Aspirational district^21^ (*n* = 1)	41	23.4	134	76.6	0.913
Non-aspirational districts (*n* = 3)	232	22.7	791	77.3	
*** Knowledge***					
Stated that VAS is beneficial	58	6.7	804	93.3	< 0.001
Able to list specific VAS benefits					
0 benefits	215	63.6	123	36.4	< 0.001
1–3 benefits	54	7.5	661	92.5	
4–6 benefits	4	2.8	141	97.2	
	**Did not receive deworming (** ***n*** ** = 304)**		**Received deworming (** ***n*** ** = 873)**		
Stated that deworming is beneficial	111	12.2	801	87.8	< 0.001
Able to list specific deworming benefits					
0 benefits	228	52.4	207	47.6	< 0.001
1–3 benefits	75	10.4	649	89.6	
4–5 benefits	1	5.6	17	94.4	

^*^Chi-square analysis

### Sociodemographic characteristics and source of VAS coverage

Cross-tabulation of sociodemographic characteristics and source of VAS received, which indicates whether the government or a CSO had provided it, showed significant associations between children taking capsule versus syrup with school enrollment (*p* < 0.001), mother’s age (*p* < 0.001), mother’s education (*p* = 0.002), mother’s occupation (*p* = 0.004), household wealth index (*p* = 0.009) and district (*p* < 0.001). Children who received the capsule form (provided by CSOs) of VAS were less likely to be enrolled in school and were less likely to have a mother who had completed high school or above. These children were also more likely to be in the lower wealth quintiles than the higher wealth quintiles and were more likely to be from the aspirational district than the non-aspirational district (Table 4).

**Table 4:** Univariate associations^*^ between demographic variables and vitamin A supplementation (VAS) source received (capsule or syrup) among children aged 6–59 months in Nagaland (*n* = 909^†^)

	Received Capsule (*n* = 649)		Received Syrup (*n* = 260)		
Child characteristics	*n*	%	*n*	%	*p*-value
**Child sex**					
Female	127	27.1	341	72.9	0.147
Male	133	30.1	308	69.8	
**Age**					
6–11 months	26	32.9	53	67.1	0.571
12–59 months	234	28.2	596	71.8	
**School enrollment status**					
No enrollment	48	76.2	15	23.8	< 0.001
Enrolled in school (i.e. *anganwadi* or other)	212	25.1	634	74.9	
***Maternal characteristics***					
**Mother’s age (years)**					
22 or lower	24	36.9	41	63.1	0.095
23–29	108	22.3	377	77.7	
30 or higher	128	35.8	230	64.3	
**Mother’s education**					
Less than high school	171	40.1	256	60.0	0.002
High school or above	89	18.5	393	81.5	
**Mother’s occupation**					
Not Working	55	15.5	299	84.5	0.004
Working	205	36.9	350	63.1	
***Household characteristics***					
**Wealth Index Quintile**					
Quintile 1 (Lowest)	13	65.0	7	35.0	0.009
Quintile 2	110	40.2	164	59.9	
Quintile 3	102	24.6	312	75.4	
Quintile 4	35	20.7	134	79.3	
Quintile 5 (Highest)	0	0.0	32	100.0	
**District**					
Aspirational district^21^ (*n* = 1)	133	99.3	1	0.8	< 0.001
Non-aspirational districts (*n* = 3)	127	16.4	648	83.6	

^*^Chi-square analysis

^†^ Those who answered “don’t know” or “both capsule and syrup” for source received were excluded from *n* (*n* = 16)

### Impact of VAS

For the LiST impact modeling, CSO VAS coverage (22%) was calculated using the participants who received the capsule form of vitamin A (*n* = 260) and the total children who received vitamin A (*n* = 1,182; those who did not know which form of Vitamin A they received were excluded from the analysis). Government vitamin A coverage (55%) was calculated using the participants who received the syrup form of vitamin A (*n* = 649). Based on LiST tool modeling [26], increased coverage of VAS through CSOs (e.g. capsule form of VAS) resulted in an estimated 9 lives saved, 114 stunting cases averted and 25,017 diarrhea cases averted in 2019.

## Discussion

The findings of the study indicate that the coverage of VAS and deworming in Nagaland increased since 2016 through combined efforts of government and CSOs, such as local NGOs. In this study, children reached by CSOs had higher odds of being from households with lower wealth quintiles, indicating that CSOs are able to decrease barriers to access among economically vulnerable populations. Furthermore, in one of Nagaland’s aspirational districts (a prioritized district by the Government of India due to low scores on indicators related to health and nutrition), VAS coverage was 69% with 99% of the VAS received from CSOs. In order to achieve the Sustainable Development Goal (SDG) of achieving universal health coverage (SDG 3.8) [27], CSOs can support the government in filling gaps in coverage by decreasing barriers to access for vulnerable populations [28, 29].

The VAS coverage of 73% found in this study exceeds the UNICEF recommended threshold of effective coverage (70%), and similarly deworming coverage (75%) meets the WHO recommended threshold (75%) [30, 31]. Despite these increases, 27% of children did not receive VAS, and this was associated with children not being enrolled in school and having mothers with less than a high school education. These findings are similar to those from a meta-analysis of data from 96 LMICs that showed that low caregiver educational status was a major barrier to childhood vaccination [32]. Similarly, a multi-country analysis of national surveys from LMICs indicates that lower maternal education is significantly associated with not receiving VAS [33].

In addition, caregivers whose children did not receive VAS were also not aware of the benefits (79%). Similar findings from studies in Kenya and India have reported that low VAS coverage due to a lack of knowledge among health workers and mothers of eligible children [34, 35]. The findings suggest that caregiver awareness needs to be addressed to increase coverage of VAS and deworming service delivery.

### Strengths and limitations

The present survey was conducted using a representative, random sample with quality control and monitoring. Another strength was different forms of VAS provided (capsule or syrup), which allowed easy determination of its source (government or CSO). One limitation was that there was a gap between VAS distribution which occurred in August 2018, and the survey which took place in December 2018, due to a flood which may have affected the respondents’ recall.

Additionally, VAS and deworming receipt was based on self-report 6-months recall data given vitamin A was delivered over many sites and times, and a same day/week post-delivery coverage survey was not possible. Moreover, at the time of this study, CSOs in Nagaland often received donated deworming medicines and often paired VAS with deworming, while government coverage of VAS was not always paired with deworming, resulting in differences in vitamin A and deworming coverage.

VAS coverage impact was not measured directly, thus there may be some uncertainty in the results modeled by LiST. However, the use of the LiST was also a strength in that it allowed us to approximate the impact of interventions that were not feasible to measure due to the limited scope and budget of the survey. Finally, given the unique partnership between CSOs and the government of Nagaland, the results may not necessarily be transferrable to other states.

### Implication for policy

This study found that collaboration between government and CSOs helped address VAS coverage gaps, contributing to decreased morbidity and mortality in children under 5. A collaborative approach that targets prioritized districts may assist in reaching more children, and in turn reduce disparities in accessing nutrition interventions, and in morbidity and mortality.

## Conclusions

Gaps in VAS and deworming coverage may be bridged toward increased coverage through innovative partnerships between government and civil society, which can ensure that the most vulnerable children are reached. Bridging such gaps results in decreased barriers toward achieving universal health coverage and reductions in illness, stunting, and death among children under five years.

### Electronic supplementary material

Below is the link to the electronic supplementary material.

Supplementary Material 1

## Data Availability

The datasets used and/or analysed during the current study are available from the corresponding author on reasonable request.

## Abbreviations

CSO: civil society organizations
LiST: Lives Saved Tool
LMIC: low- and middle- income countries
VAD: vitamin A deficiency
VAS: vitamin A supplementation
VAS + D: vitamin A supplementation and deworming
