# Supplementary material for: Bridging the vitamin A and deworming coverage gap among underserved populations in India through government and civil society organization partnerships
Source: Arch Public Health. 2024 May 20;82:75. doi: 10.1186/s13690-024-01302-8 (PMC11103955; doi:10.1186/s13690-024-01302-8)
Supplement: Supplementary file 1 — Supplementary Material 1 [file 13690_2024_1302_MOESM1_ESM.docx]

**Bridging the vitamin A and deworming coverage gap among underserved populations in India through government and civil society organization partnerships**

**Supplementary Table 1. Univariate associations^*^ between demographic variables and retention status (retained or dropped) among children aged 6-59 months in Nagaland (n=1272)**

|  | **Children retained (n=1198)** | | **Children dropped (n=74)** | |  |
| --- | --- | --- | --- | --- | --- |
| ***Child characteristics*** | ***n*** | **%** | ***n*** | **%** | **p-value** |
| **Child sex** |  |  |  |  |  |
| Female | 617 | 94.8 | 32 | 5.2 | 0.383 |
| Male | 581 | 93.6 | 42 | 6.4 |  |
| **Age** |  |  |  |  |  |
| 6-11 months | 158 | 92.4 | 13 | 7.6 | 0.688 |
| 12-59 months | 1040 | 94.5 | 61 | 5.5 |  |
| **School enrollment status** |  |  |  |  |  |
| No enrollment | 133 | 94.3 | 8 | 5.7 | 0.951 |
| Enrolled in school (i.e. *anganwadi* or other) | 1065 | 94.2 | 66 | 5.8 |  |
| ***Maternal characteristics*** |  |  |  |  |  |
| **Mother’s age (years)** |  |  |  |  |  |
| 22 or lower | 106 | 94.6 | 6 | 5.4 | 0.317 |
| 23-29 | 609 | 95.0 | 32 | 5.0 |  |
| 30 or higher | 482 | 93.1 | 36 | 6.9 |  |
| **Mother’s education** |  |  |  |  |  |
| Less than high school | 597 | 93.7 | 40 | 6.3 | 0.643 |
| High school or above | 601 | 94.7 | 34 | 5.4 |  |
| **Mother’s occupation** |  |  |  |  |  |
| Not Working | 472 | 92.9 | 36 | 7.1 | 0.281 |
| Working | 726 | 95.0 | 38 | 5.0 |  |
| ***Household characteristics*** |  |  |  |  |  |
| **Wealth Index Quintile** |  |  |  |  |  |
| Quintile 1 (Lowest) | 25 | 80.7 | 6 | 19.3 | 0.218 |
| Quintile 2 | 357 | 94.4 | 21 | 5.6 |  |
| Quintile 3 | 550 | 93.7 | 37 | 6.3 |  |
| Quintile 4 | 228 | 95.8 | 10 | 4.2 |  |
| Quintile 5 (Highest) | 38 | 100.0 | 0 | 0.0 |  |
| **District** |  |  |  |  |  |
| Aspirational district^21^ (n=1) | 1023 | 94.9 | 55 | 5.1 | 0.076 |
| Non-aspirational districts (n=3) | 175 | 90.2 | 19 | 9.8 |  |

^*^Chi-square analysis.
